# Implications for Clinical Practice from a Multicenter Survey of Heart Failure Management Centers

**DOI:** 10.6061/clinics/2021/e1991

**Published:** 2021-01-11

**Authors:** Edimar Alcides Bocchi, Henrique Turin Moreira, Juliana Sanajotti Nakamuta, Marcus Vinicius Simões, CLIMB-HF Study Group, Alberto de Almeida Las Casas, Altamiro Reis da Costa, Amberson Vieira de Assis, André Rodrigues Durães, Antonio Carlos Pereira-Barretto, Antonio Delduque de Araujo Ravessa, Ariane Vieira Scarlatelli Macedo, Bruno Biselli, Carolina Maria Nogueira Pinto, Conrado Roberto Hoffmann Filho, Costantino Roberto Costantini, Dirceu Rodrigues Almeida, Edval Gomes dos Santos, Erwin Soliva, Estevão Lanna Figueiredo, Felipe Neves de Albuquerque, Felipe Paulitsch, Fernando Carvalho Neuenschwander, José Albuquerque de Figueiredo, Flavio de Souza Brito, Heno Ferreira Lopes, Humberto Villacorta, João David de Souza, João Mariano Sepulveda, José Carlos Aidar Ayoub, José F. Vilela-Martin, Juliano Novaes Cardoso, Laercio Uemura, Lidia Zytynski Moura, Lilia Nigro Maia, Lucia Brandão de Oliveira, Lucimir Maia, Luís Beck da Silva, Luís Henrique Wolff Gowdak, Luiz Claudio Danzmann, Marcus Andrade, Maria Christiane Valeria Braga Braile-Sternieri, Maria da Consolação Vieira Moreira, Olimpio R França, Otavio Rizzi Coelho Filho, Paulo Frederico Esteves, Priscila Raupp-da-Rosa, Ricardo Jorge de Queiroz e Silva, Ricardo Mourilhe-Rocha, Ruy Felipe Melo Viégas, Salvador Rassi, Sandrigo Mangili, Sergio Emanuel Kaiser, Silvia Marinho Martins, Vitor Sergio Kawabata

**Affiliations:** I Nucleo de Insuficiencia Cardiaca, Instituto do Coracao (InCor), Hospital das Clinicas HCFMUSP, Faculdade de Medicina, Universidade de Sao Paulo, Sao Paulo, SP, BR; II Faculdade de Medicina de Ribeirao Preto, Universidade de Sao Paulo, Ribeirao Preto, SP, BR; III Novartis AG, Basel, Switzerland

**Keywords:** Heart Failure, Disease Management Program, Education Monitoring, Clinical Decision-Making, Multidisciplinary Treatment

## Abstract

**OBJECTIVES:**

This observational, cross-sectional study based aimed to test whether heart failure (HF)-disease management program (DMP) components are influencing care and clinical decision-making in Brazil.

**METHODS:**

The survey respondents were cardiologists recommended by experts in the field and invited to participate in the survey via printed form or email. The survey consisted of 29 questions addressing site demographics, public *versus* private infrastructure, HF baseline data of patients, clinical management of HF, performance indicators, and perceptions about HF treatment.

**RESULTS:**

Data were obtained from 98 centers (58% public and 42% private practice) distributed across Brazil. Public HF-DMPs compared to private HF-DMP were associated with a higher percentage of HF-DMP-dedicated services (79% *vs* 24%; OR: 12, 95% CI: 94-34), multidisciplinary HF (MHF)-DMP [84% *vs* 65%; OR: 3; 95% CI: 1-8), HF educational programs (49% *vs* 18%; OR: 4; 95% CI: 1-2), written instructions before hospital discharge (83% *vs* 76%; OR: 1; 95% CI: 0-5), rehabilitation (69% *vs* 39%; OR: 3; 95% CI: 1-9), monitoring (44% *vs* 29%; OR: 2; 95% CI: 1-5), guideline-directed medical therapy-HF use (94% *vs* 85%; OR: 3; 95% CI: 0-15), and less B-type natriuretic peptide (BNP) dosage (73% *vs* 88%; OR: 3; 95% CI: 1-9), and key performance indicators (37% *vs* 60%; OR: 3; 95% CI: 1-7). In comparison to non- MHF-DMP, MHF-DMP was associated with more educational initiatives (42% *vs* 6%; OR: 12; 95% CI: 1-97), written instructions (83% *vs* 68%; OR: 2: 95% CI: 1-7), rehabilitation (69% *vs* 17%; OR: 11; 95% CI: 3-44), monitoring (47% *vs* 6%; OR: 14; 95% CI: 2-115), GDMT-HF (92% *vs* 83%; OR: 3; 95% CI: 0-15). In addition, there were less use of BNP as a biomarker (70% *vs* 84%; OR: 2; 95% CI: 1-8) and key performance indicators (35% *vs* 51%; OR: 2; 95% CI: 91,6) in the non-MHF group. Physicians considered changing or introducing new medications mostly when patients were hospitalized or when observing worsening disease and/or symptoms. Adherence to drug treatment and non-drug treatment factors were the greatest medical problems associated with HF treatment.

**CONCLUSION:**

HF-DMPs are highly heterogeneous. New strategies for HF care should consider the present study highlights and clinical decision-making processes to improve HF patient care.

## INTRODUCTION

Heart failure (HF) is the leading cause of hospital admission and readmissions in the United States among adults aged ≥65 years, creating a significant healthcare burden (1). Despite the undeniable progress in HF treatment over the past few years, the number of hospital readmissions and associated healthcare costs remain very high (2). The goals of the HF disease management programs (DMPs) or HF clinics, include optimization of drug therapy, intensive patient education, vigilant follow-up for early recognition of complications, and identification and management of patients’ comorbidities to reduce mortality rates, hospital admissions, and improve patients’ health-related quality of life (2-4).

HF-DMPs are associated with improved HF outcomes (5). However, the content and effectiveness of HF-DMP interventions may vary widely (6) since they contain several components that contribute to their success (7). However, in clinical practice, the association of each component with the effectiveness of the HF-DMP has not been studied and HF care models in low- and middle-income countries remain unknown. In addition, doctor perceptions about the decision-making process in HF management have not been previously reported. Therefore, this study aimed to evaluate HF care models and HF-DMPs in a middle-income country using a questionnaire-based cross-sectional survey in a clinical setting.

## METHODS

### Survey design

The CLIMB-HF is an observational, cross-sectional, survey-based study administered to cardiologists from major cardiology centers across Brazil. The survey was created to gather information regarding HF-DMP and/or HF care models routinely used in clinical practice.

### Eligibility

Participants were recommended by members of the Heart Failure Department of the Brazilian Society of Cardiology based on their expertise and active engagement in HF patient management. The participant selection also aimed to include a broad range of public and private cardiology practice settings. Public institutions were defined as centers in which the government was responsible for patient management and related costs. Private centers were defined by financial support from private insurance companies covering a pre-defined list of medical procedures or by the patient.

### Heart failure care model questionnaire

After reviewing the literature on DMPs for HF patients and in HF clinics, a questionnaire was developed with the assistance of expert cardiologists from clinics with HF-DMPs (Supplementary Table S1). All the physicians were also members of the Department of Heart Failure from the Brazilian Society of Cardiology. Patient chart review was not performed for this study. The interviewed physicians provided answers based on their perceptions and conceptions. The questionnaire (either in physical form or electronic using the SurveyMonkey platform) was distributed by the Novartis team from June to October 2016 to selected cardiologists, both from public and private systems. The survey was sent as a printed questionnaire or by email in an electronic format. The answers were anonymous. For some questions, the participants were asked to submit responses in order of importance. The questionnaire consisted of 29 questions to collect demographic data regarding the participant’s work facility; the type of facility (private or public); HF patient characteristics according to the physicians’ perception; HF clinical management including practice during follow-up, educational programs for patients and caregivers, monitoring, and/or cardiac rehabilitation; and performance indicators (Appendix).

### Objectives

The primary objective of the study was to evaluate the status of HF care models implemented in cardiology centers in Brazil.

The secondary objectives were to report the characteristics of the HF care models including implementation of educational programs for patients and caregivers, monitoring and cardiac rehabilitation programs, multidisciplinary team components, clinical practice in the management of HF patients under HF care models, demographic data and structure of the participating centers, the profile of HF patients, utilization of performance indicators related to HF, and cardiologists’ perceptions regarding patient profiles and treatment standards.

We compared private and public setting characteristics (the type of infrastructure and function) of HF care defined by subspecialized HF treatment available in ambulatory care, subspecialized HF treatment available hospitalization, and multidisciplinary care.

### Data analysis

The center identities were blinded during the data analysis. Categorical variables are reported as percentages and numbers. Continuous data are presented as mean±standard deviation if normally distributed, or as the median and interquartile range if non-normally distributed. Groups were defined according to health care setting (public *versus* private), availability of specialized service for HF, higher specialized HF service, and multidisciplinary care. Centers with higher specialization for HF care were defined as those with a multidisciplinary approach with at least a cardiologist, a nurse, a physiotherapist (or physical educator), and a nutritionist. Those with partial or without multidisciplinary teams were considered non-specialized. Chi-square and Fisher’s exact tests were used to compare categorical variables between groups, as appropriate. Variables obtained as estimate proportions were compared using a test on equality of proportions after estimating counts based on patients in follow-up for each variable. All analyses were two-tailed and conducted at the 5% significance level. The sample size was not pre-specified, but a good representation of the different regions of Brazil was sought.

## RESULTS

The questionnaire was sent as a printed questionnaire to 121 cardiologists (50 replied) and to 184 cardiologists in an electronic format with a link for the same questionnaire in the SurveyMonkey platform (49 replied). The total number of completed questionnaires received was 99, and one questionnaire that did not specify the type of service (public or private) was excluded from the analysis. Among the 98 valid questionnaires, 57 (58%) came from private centers and 41 (42%) came from public centers.

### Demographic and structure data from participating sites

Demographic and site characteristics are summarized in Table 1. In both private and public centers, the most common HF etiology was ischemia. The availability of implantable cardioverter defibrillators (ICD)/cardiac resynchronization therapy (CRT), surgical treatment, multidisciplinary care, cardiologists, cardiac surgeons, nurses, nutritionists, physiotherapists, psychologists, pharmacists, and physical trainers was reported. Results showed important differences between public and private center characteristics. In the public centers, the number of patients was higher with greater presentation of severe cases according to New York Heart Association functional class. Ischemic/hypertensive/valvular etiologies were more frequent in patients treated in the private setting, while patients treated in public centers more often presented with chagasic and idiopathic dilated cardiomyopathy. The percent of possible available treatments, presence of multidisciplinary care, and its components were higher in the public care system.

### Delivered care for heart failure patients in participating centers

B-type natriuretic peptide (BNP) or N-terminal pro-brain natriuretic peptide (NT-proBNP) is available in most centers and is used especially for diagnosis and prognosis. Educational programs were generally underused, except in the public care system. Nonetheless, written instructions at hospital discharge were frequently used and performed by doctors and/or nurses in both settings. The participation rates in monitoring programs were low in both systems (public and private) but were higher in the public setting. The regular use of guidelines was very high in both public and private care systems, and the Brazilian Guidelines were the most utilized. However, other international guidelines were also used. Quality of life questionnaires were not conducted in most centers. Rehabilitation programs were more frequently available in the public care system. Key performance indicators were mainly used by private care centers. During hospitalization, medical decisions were mostly made by the hospital’s cardiology team or intensive care unit physicians. In private care, the outpatient unit cardiologists were responsible for the majority of HF treatment initiation, while in public care, this was performed by both the hospital cardiology team and outpatient facilities. There was no difference in the use of standard guideline-oriented therapy for HF, except for lower use of digoxin in private care. In public settings, compliance with the target dose of medications according to guidelines was higher.

When comparing the characteristics of care according to the presence or absence of a multidisciplinary team, there was a higher rate of BNP or NT-proBNP use to support treatment decisions, increased rates of educational, monitoring and rehabilitation programs, and a higher proportion of patients on target doses of medications according to guidelines (Table 2 and Supplementary Table S2). Conversely, in centers without multidisciplinary care, medical decisions were performed more frequently by the hospital cardiology team. In centers with HF subspecialized care, there was a higher percentage of patient monitoring programs mainly in outpatient units, in addition to the use of non-doctor-specialized monitoring methods, digoxin, and a higher proportion of patients on target doses of medications according to the guidelines (Supplementary Table S3). In centers with highly specialized multidisciplinary care, the results also showed a greater percentage of patient monitoring programs at outpatient clinics using non-doctor specialized monitoring programs, more cardiac rehabilitation, higher use of digoxin/vasodilators, and patients under the target dose of medication according to guidelines (Supplementary Table S4).

### Evaluation of medical expectations, treatment objectives, and challenges in HF management

**Figure 1:**
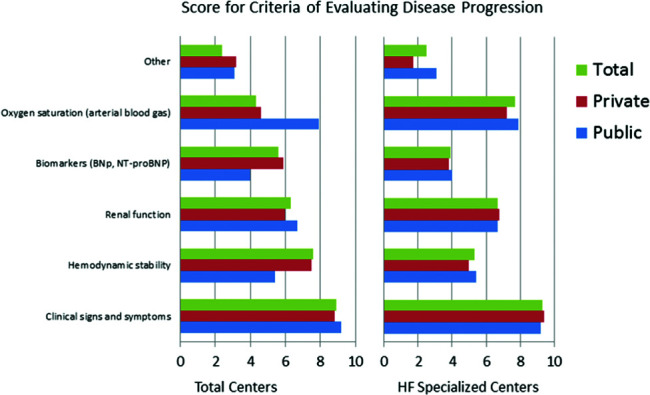
Perceptions of doctors in centers treating heart failure (HF) regarding a criteria for evaluation of HF disease progression using a score in total, private, and public HF patients in HF specialized and all centers.

**Figure 2:**
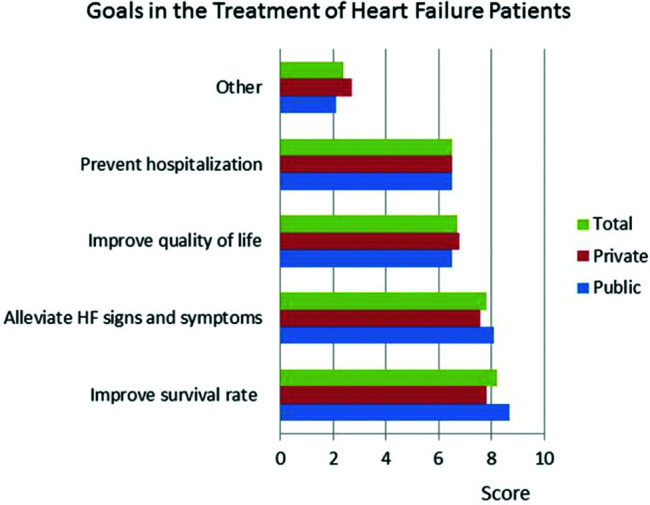
Perceptions of doctors in centers treating heart failure (HF) patients about goals of HF treatment using a score for total, private, and public settings.

**Figure 3:**
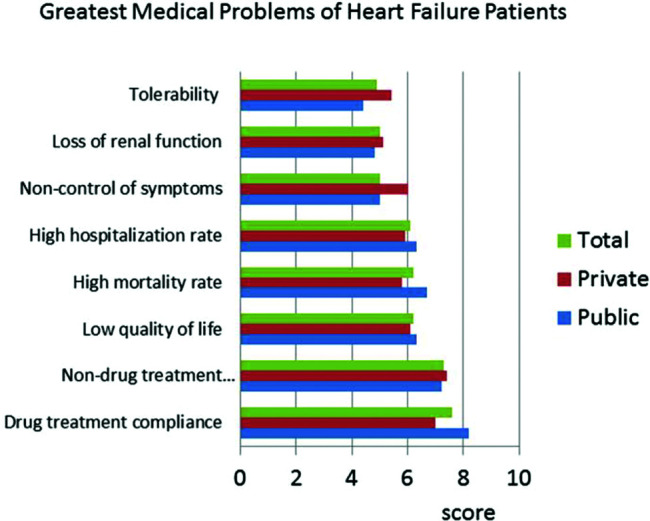
Perceptions of doctors in centers treating heart failure (HF) regarding the greatest medical problems of HF patients using a score for total, private, and public settings.

**Figure 4:**
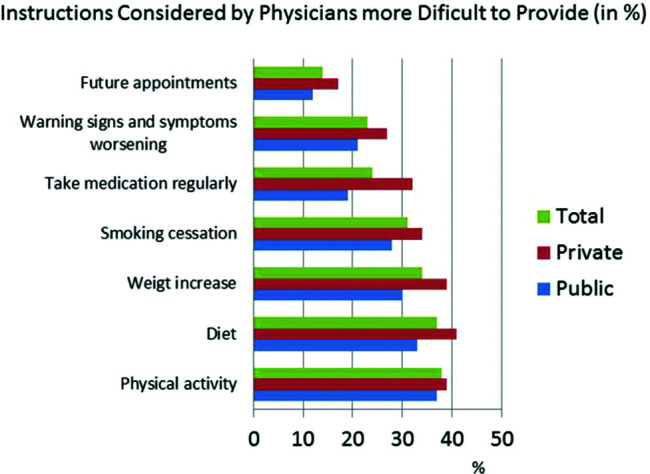
Perceptions of doctors in centers treating heart failure (HF) regarding instructions considered more difficult to provide according to percent in total, private, and public settings.

**Figure 5:**
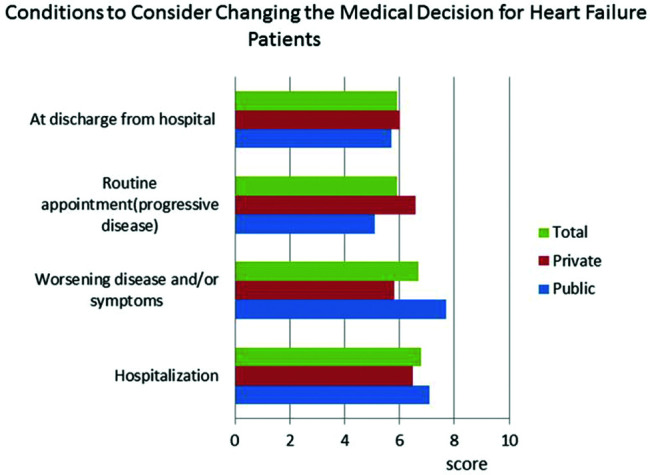
Perceptions of doctors in centers treating heart failure (HF) regarding the importance of conditions that lead to change in the management of HF patients.

The responses to the question &quot;What are the most important criteria for evaluating disease progression?&quot; were ranked by level of importance by the respondents. The results showed that in both public and private centers, clinical signs and symptoms received the highest scores, followed by hemodynamic instability (Figure 1). BNP was less important in centers with an HF specialized team. Questions about HF treatment goals showed that survival, symptoms, quality of life, and hospitalization were important in descending order (Figure 2). Drug treatment compliance was the greatest medical challenge in treating HF patients (Figure 3). The instructions considered by physicians as the most difficult to provide according to highest to lowest percent were those for physical activity, diet, prevention of weight gain, and smoking cessation (Figure 4). Difficulties with compliance were more frequent in private settings patients. Overall, hospitalization, worsening of symptoms, routine appointments, and hospital discharge were considered as signs for treatment optimization (Figure 5).

## DISCUSSION

To the best of our knowledge, our results are the first summary of HF-DMP characteristics from cardiology centers involved in the management of HF patients in Brazil. We found that the heterogeneity of HF-DMPs may affect the care delivered. HF patients in public care centers had a different profile compared to patients in private care concerning the severity and etiology of their disease. Educational programs and multidisciplinary components were more frequently reported in public care settings. In private centers, hospitalization and 6-month mortality rates were the most frequently used key performance indicators. In the private care system, ambulatory cardiologists were responsible for most medical decisions and HF treatment initiation in hospitalized patients, whereas in public centers, the hospital cardiology team made most decisions. Limitations concerning the patient instructions were reported. There was no consensus about the best moment to consider changing methods for HF management.

Public and private care coexist in many countries around the world. Unfortunately, there is no published data on HF treatment in private and public care in the same country to compare our results. One explanation for the greater HF case severity in patients treated in public centers, along with a higher prevalence of Chagas disease, might be the low income and restricted access to cardiology care. The higher prevalence of more severe cases of HF in public centers may demand more surgical treatments and multidisciplinary care components. Multidisciplinary programs may be more effective in treating high-risk patients and, consequently, more reasonable in public settings (8). From an economic point of view, the lack of reimbursement may be a limiting factor for the existence of multidisciplinary teams in private settings.

The heterogeneity of HF-DMPs in the present study is similar to previously reported data (8). A vast range of combinations regarding HF care models have been described, varying from a single educational session before discharge; a single educational home visit by a nurse specialist and regular telephone follow-up; or a multidisciplinary intervention centralized in a dedicated facility, with or without primary care interaction (4).

The reported percentage of HF educational programs for patients and caregivers in private centers is considered low when compared to published data from selected countries (9). Accordingly, the percentage of nurses who were the main providers of patient education in inpatient and outpatient settings was lower in private care (10). In the present study, the observed percentage of educational programs for caregivers was even lower than that from published data, both for public and private centers. It is conceivable that this may be associated with poorer clinical outcomes since caregivers contribute to HF patient care and management but without proper education, some of their practices are not evidence-based (11). Conversely, caregiver training for early recognition of symptoms and signs of worsening HF may be effective in reducing hospitalizations (12).

The low use of non-invasive monitoring programs (home monitoring or telephone interviews) in both private and public care systems could be explained by the mixed and controversial evidence, which show poor efficacy for reducing serious negative outcomes in HF (13). On the other hand, we observed higher rates of HF instructions at hospital discharge, despite inconsistent or controversial results from previous studies (14). It is possible that instructions and follow-up planning at discharge are tailored according to the characteristics of each institution and patient population, creating more effective follow-up. In addition, instructions and planning at discharge are not too complex to be implemented in clinical practice.

We observed a non remarkable rate of cardiac rehabilitation availability (54% of centers), which was higher in public centers (69% in public centers *versus* 39% in private centers, *p*=0.016). These data probably reflect the current recommendations (15), despite the neutral results of the HF-ACTION trial on mortality which were corroborated by a recent meta-analysis (16-19). However, according to the updated Cochrane review, exercise-based rehabilitation reduces the risk of hospital admissions and confers important improvements in health-related quality of life. However, it is important to consider that the availability of rehabilitation services does not mean that a high percentage of patients are actually under rehabilitation because the adherence rate to exercise is usually modest (20). In addition, our survey showed that instructions for exercise were one of the most difficult to provide. This is an important point, as previous studies have reported that one-third of HF patients had a low level of physical activity in their daily life (21). Furthermore, only a small number of the participating centers used a quality of life assessment to evaluate treatment success.

One positive result from the present study was that the prescribed HF treatment and its goals are in accordance with guideline-directed medical therapy for HF (GDMT-HF) (22). Unfortunately, the data also showed that physicians mostly consider changing or introducing new medications when patients are hospitalized or when diagnosis indicates worsening disease and/or symptoms. These findings might partly explain why in a contemporary US registry, most eligible HF patients with reduced ejection fraction did not receive target doses of medical therapy at any time point during follow-up, and few patients had doses increased over time (23).

### Limitations

This study has several limitations, many that are typical limitations of using a survey for data collection (24). Despite these limitations, surveys are very important tools to understand the current practice of medicine around the world. This study may be limited by its cross-sectional nature and therefore could be biased in its selection of centers, sampling approaches, and variables, which could overestimate or underestimate true values. The study population was limited to selected cardiologists belonging to HF centers and did not include general practitioners or other physicians involved in the care of HF patients. Other limitations include the limited generalizability of the results, since the study was conducted in selected centers. No data monitoring was performed since the survey was self-reported. Significant results from this analysis should be confirmed in prospective studies. In addition, recent advances in the pharmacological treatment of HF, such as angiotensin receptor-neprilysin inhibitor (ARNI) (*i.e.* valsartan/sacubitril), dapaglifozine, and sinoatrial node modulators (ivabradine) were not included in this survey (25,26). Data supporting answers for the questionnaire were not collected; however, this is a standard limitation for self-reported surveys. The survey answers were anonymous to reduced response-bias and encourage responders to provide accurate and honest answers since they may not feel comfortable unfavorably presenting themselves.

Despite these limitations, the strength of this study includes the stratification of HF data representing the daily clinical practice of HF management in Brazil.

## CONCLUSIONS

HF-DMPs are heterogeneous, with many components still underutilized in private practice. The present survey extends our understanding of HF management in Brazil. Based on our study, strategies can be developed to improve outcomes in patients with HF. In addition, the characteristics of HF centers can be influenced, based on whether they are in a private or public setting. From an optimistic perspective, our results demonstrate that HF patient care is established on an evidence-based approach, attempting to ensure the achievement of pharmacological GDMT goals independent of the center setting. However, the characteristics of non-pharmacological management are heterogeneous despite GDMT-HF. The diverse features of the HF-MDPs in multiple centers managing HF could partially reflect the lack of consistent data or controversial results from prospective trials, mainly for educational and monitoring programs, multidisciplinary team components, and rehabilitation.

The findings demonstrate that physicians mostly consider shifting or introducing new therapies when patients are hospitalized or the symptoms are striking. Thus, there is an evident need for improvements in education for both physicians and patients.

### Highlights

- In general, the components of DMPs in heart failure (HF) clinics are heterogeneous.
- Non-private HF and multidisciplinary care were associated with a higher percentage of dedicated service to HF, education, and guideline-directed medical therapy for HF treatment.
- Physicians considered changing or introducing new medications mostly when patients were hospitalized or when diagnosis indicated disease and/or symptoms worsening.
- Adherence to drug treatment and non-drug treatment were the greatest medical challenges associated with HF treatment.

The planning of new strategies for HF care should consider DMP-HF characteristics and clinical decision-making processes to improve HF patient care.

**Conflicts of Interest**. Edimar Alcides Bocchi: Consulting Fee: Servier, Astra-Zeneca; Subsidized Travel/Hotel/Registration Fees: Servier; Membership in Steering Committee: Servier, Novartis; Contracted Research: Jansen, Bayer/Merck; Honoraria: Novartis; Henrique Turin Moreira, nothing to declare; Juliana Sanajotti Nakamuta; Novartis employees; Marcus Vinicius Simões; Novartis, Boehringer Ingelheim, Honoraria Novartis, Servier, EMS.

### CLIMB-HF study investigators (in alphabetical order). Collaborators who agreed to be mentioned and included in the CLIMB-HF Study Group (according to information obtained by Novartis):

Alberto de Almeida Las Casas (Hospital do Coração Anis Rassi), Altamiro Reis da Costa (Instituto de Cardiologia do Rio Grande do Sul), Amberson Vieira de Assis (Instituto de Cardiologia de Santa Catarina), André Rodrigues Durães(Hospital Ana Nery/UFBa), Antonio Carlos Pereira-Barretto (Instituto do Coração - InCor-SP), Antonio Delduque de Araujo Ravessa (Centro Médico), Ariane Vieira Scarlatelli Macedo (Rede Mater Dei de Saúde), Bruno Biselli (Hospital Sírio-Libanês), Carolina Maria Nogueira Pinto (Total Care - AMIL - São Paulo), Conrado Roberto Hoffmann Filho (Hospital Regional Hans Dieter Schimidt), Costantino Roberto Costantini (Hospital Cardiológico Costantini), Dirceu Rodrigues Almeida (Universidade Federal de São Paulo (UNIFESP) - Hospital São Paulo), Edval Gomes dos Santos Jr (Hospital Dom Pedro de Alcântara), Erwin Soliva Junior (Hospital Universitário do Oeste do Paraná), Estevão Lanna Figueiredo (Hospital Lifecenter - Belo Horizonte -MG), Felipe Neves de Albuquerque (Hospital das Clínicas da Universidade do Estudo do Rio de Janeiro), Felipe Paulitsch (Universidade Federal do Rio Grande), Fernando Carvalho Neuenschwander (Hospital Vera Cruz - Belo Horizonte), Jos� Albuquerque de Figueiredo Neto (Universidade Federal do Maranhão), Flavio de Souza Brito (Total Care - AMIL - São Paulo), Heno Ferreira Lopes (Instituto do Coração - InCor-SP), Humberto Villacorta (Universidade Federal Fluminense), João David de Souza Neto (Hospital de Messejana Dr. Carlos Alberto Studart Gomes), João Mariano Sepulveda (Instituição Medicare), José Carlos Aidar Ayoub (Instituto de Moléstias Cardiovasculares Rio Preto Ltda), José F. Vilela-Martin (Faculdade de Medicina de São José do Rio Preto (FAMERP), Juliano Novaes Cardoso (Hospital Santa Marcelina), Laercio Uemura (Universidade Estadual de Londrina, Centro do Coração de Londrina), Lidia Zytynski Moura (Santa Casa de Curitiba - PUCPR), Lilia Nigro Maia (Hospital de Base/Faculdade de Medicina de Rio Preto (FAMERP), Lucia Brandão de Oliveira (Clínica de Insuficiência Cardíaca do Centro Universitário Serra dos =rgãos (UNIFESO)), Lucimir Maia (Hospital Regional do Guará), Luís Beck da Silva (Hospital de Clínicas de Porto Alegre), Luís Henrique Wolff Gowdak (Instituto do Coração - InCor-SP), Luiz Claudio Danzmann (Universidade Luterana do Brasil), Marcus Andrade (Hospital Santa Izabel - Santa Casa de Misericórdia da Bahia), Maria Christiane Valeria Braga Braile-Sternieri (Instituto Domingo Braile), Maria da Consolação Vieira Moreira (Hospital das Clínicas da UFMG), Olimpio R. França Neto (Instituto Paranaense de Cardiologia), Otavio Rizzi Coelho Filho (Hospital das Clínicas da UNICAMP), Paulo Frederico Esteves (Hospital Santa Mônica, Divinópolis, MG); Priscila Raupp-da-Rosa (Hospital Divina Providência), Ricardo Jorge de Queiroz e Silva (Universidade Federal do Rio Grande do Norte), Ricardo Mourilhe-Rocha (Universidade do Estado do Rio de Janeiro), Ruy Felipe Melo Viégas (Universidade de Taubaté (UNITAU)), Salvador Rassi (Hospital das Clínicas da Universidade Federal de Goiás), Sandrigo Mangili (Hospital Municipal da Vila Santa Catarina), Sergio Emanuel Kaiser (Hospital Universitário Pedro Ernesto), Silvia Marinho Martins (Ambulatório de Doença de Chagas e Insuficiência Cardíaca - PROCAPE - Universidade de Pernambuco), Vitor Sergio Kawabata (Hospital Universitário da Universidade de São Paulo).

## AUTHOR CONTRIBUTIONS

Bocchi EA was responsible for the study conception and design, data analysis and interpretation, and manuscript drafting and review. Moreira HT was responsible for the data analysis and interpretation, and manuscript drafting and review. Nakamuta JS was responsible for the acquisition of data, study conception and design, data analysis and interpretation, and manuscript drafting and review. Simões MV was responsible for the data analysis and interpretation, and manuscript drafting and review. All authors approved the final version of the manuscript.

## Figures and Tables

**Table 1:** Demographic and Structure Data from Participating Sites Comparing Public *versus* Private.

	Total n=99*	Public n=41	Private n=57	*p*-value
Patients under follow-up	200 [100-600]	500 [200-950]	180 [50-130]	<0.001
Patients attended per week	16 [7-40]	40 [16-60]	10 [2-20]	<0.001
NYHA functional class (%)				
I	18%	14%	28%	<0.001
II	36%	34%	43%	<0.001
III	29%	32%	20%	<0.001
IV	17%	20%	9%	<0.001
Etiology (%)				
Ischemic	29%	28%	32%	<0.001
Hypertensive	18%	16%	22%	<0.001
Valvular	10%	7%	16%	<0.001
Chagasic	15%	18%	9%	<0.001
Cardiotoxicity	2%	2%	2%	ns
Peripartum	3%	3%	3%	ns
Idiopathic	17%	21%	9%	<0.001
Others	6%	5%	7%	0.06
Number of hospitalizations due to HF per month	10 [4-20]	20 [10-25]	10 [2-15]	0.001
Outpatient care type				
General care	10% (9/90)	5% (2/38)	12% (5/51)	<0.001
Cardiology care	55% (39/90)	16% (6/38)	65% (33/51)	
Outpatient HF care	18% (16/90)	21% (8/38)	16% (8/51)	
Outpatient and inpatient HF care	29% (26/90)	58% (22/38)	8% (4/51)	
Available treatments				
ICD and CRT	72% (63/87)	87% (33/38)	65% (30/49)	0.008
Surgical	77% (67/87) (67 out of 87)	95% (36/38) (36 out of 38)	63% (31/49) (31 out of 49)	0.001
Heart transplant	31% (27/87)	45% (17/38)	20% (10/49)	0.015
Circulatory support	76% (39/87)	58% (22/38)	35% (17/49)	0.031
Multidisciplinary care	74% (64/87)	84% (31/37)	65% (22/49)	0.055
Multidisciplinary components				
Cardiologists	94% (59/63)	100% (31/31)	88% (28/32)	0.060
Cardiac Surgeon	76% (51/63)	94% (29/31)	59% (19/32)	0.002
Nephrologists	56% (35/63)	66% (21/31)	44% (14/32)	0.055
Nurses	86% (55/63)	100% (31/31)	75% (24/32)	0.005
Nutritionists	80% (51/63)	87% (27/31)	75% (24/32)	ns
Physiotherapists	70% (45/63)	81% (25/31)	63% (20/32)	ns
Psychologists	67% (43/63)	87% (27/31)	50% (16/16)	0.002
Social workers	62% (40/63)	88% (28/31)	38% (12/32)	<0.001
Pharmacists	53% (34/64)	61% (19/31)	47% (15/47)	ns
Physical trainer	50% (32/64)	55% (17/31)	47% (15/47)	ns
Others	53% (34/64)	7% (2/31)	9% (3/32)	ns
Number of multidisciplinary components by center	8 [6-10]	9 [7-10]	7 [4-10]	0.011

IQR, interquartile range; HF, heart failure; ICD, implantable cardioverter defibrillators; CRT, cardiac resynchronization therapy; NYHA, New York Heart Association.

Multidisciplinary centers have at least two multidisciplinary members of the following professions: cardiology, nursing, physiotherapy and/or physical educator or nutritionist.

* one center did not state type of center; *p-*value for public *versus* private.

**Table 2:** Delivered Care for Heart Failure Patients Comparing Public *versus* Private.

	Total	Public	Private	*p-*value
BNP dosage available	81% (64/79)	73% (27/37)	88% (37/42)	0.087
Indication for BNP				
Help in the diagnosis	72% (46/64)	67% (18/27)	75% (28/37)	ns
Determine the prognosis	61% (39/64)	63% (17/27)	60% (22/37)	ns
Help the treatment	33% (6/27)	22% (6/27)	41% (15/37)	ns
Monitoring	58% (37/64)	48% (13/27)	65% (24/37)	ns
Centers without BNP				
Lack of access	80% (13/15)	80% (8/10)	100% (5/5)	ns
Considered not essential	20% (3/15)	30% (3/10)	0% (0/5)	ns
Education program for patient	33% (24/73)	55% (17/31)	22% (7/32)	0.006
Education program for caregivers	24% (18/74)	39% (12/31)	19% (6/32)	0.058
Written instructions at hospital discharge	84% (63/75)	83% (29/35)	76% (29/38)	ns
By Doctor	66% (38/58)	62% (18/29)	69% (20/29)	ns
By Nurse	33% (19/58)	38% (11/29)	28% (8/29)	
By Others	1% (1/58) (1 out of 58)	0% (0/29) (0 out of 29)	3% (1/29) (1 out of 29)	
Patient monitoring programs	39% (27/70)	47% (17/36)	29% (10/34)	ns
Using monitoring program				
At ambulatory using non-doctorspecialized monitoring	20% (14/70)	31% (11/36)	9% (3/34)	0.035
At distance using phone or other methods	14% (10/70)	14% (5/36)	15% (5/34)	ns
Regular use of HF guidelines	90% (65/72)	94% (34/36)	86% (31/36)	ns
DEIC-BSC	43% (31/72)	50% (18/36)	36% (13/36)	ns
ESC	21% (15/72)	19% (7/36)	22% (8/36)	
AHA/ACC	17% (12/72)	14% (5/36)	16% (7/36)	
Institution’s own protocol	8% (6/72)	8% (3/36)	8% (3/36)	
Other guidelines	1% (2/72)	3% (1/36)	0% (0/36)	
Not following guidelines	10% (7/72)	6% (2/36)	14% (2/36)	
Using quality of life questionnaire	26% (18/69)	23% (8/35)	29% (10/34)	ns
KCCQ	3% (2/69)	3% (1/35)	3% (1/34)	ns
MLHFQ	20% (14/69)	17% (6/35)	24% (8/34)	ns
Other	3% (2/69)	3% (1/35)	3% (1/34)	ns
Cardiac rehabilitation	54% (37/68)	69% (24/35)	39% (13/33)	0.016
Key performance indicators	53% (34/64)	39% (13/33)	68% (21/31)	0.023
Hospital mortality	31% (20/64)	22% (7/33)	42% (13/31)	0.074
6-month mortality	20% (13/64)	18% (6/33)	23% (7/31)	ns
30-day hospitalization after discharge	33% (21/64)	24% (8/33)	42% (13/31)	ns
90-day hospitalization after discharge	14% (9/64)	12% (4/33)	16% (5/31)	ns
Hospitalization duration	25% (6/64)	24% (8/33)	26% (8/31)	ns
Other	3% (2/64)	3% (1/33)	3% (1/31)	ns
Medical decision in hospitalized patient in decompensated HF				
By hospital cardiology team	44%	48%	38%	< 0.001
By cardiologist who cares for the patient in the outpatient clinic	43%	41%	46%	< 0.001
By intensive care unit doctors if admitted in intensive care unit	13%	11%	16%	< 0.001
Beginning of HF treatment				
Cardiologist in hospital	38%	45%	27%	< 0.001
Cardiologist in ambulatory	43%	30%	62%	< 0.001
General practitioner	11%	15%	4%	< 0.001
Intensive care doctor	4%	3%	5%	0.102
Geriatrics	2%	3%	0.4%	0.276
Others	3%	4%	2%	0.147
Prescribed HF treatment				
ACE-I	97% (72/74)	92% (33/36)	100% (38/38)	ns
ARBs	84% (62/74)	81% (29/36)	87% (33/38)	ns
β-blocker	92% (68/74)	92% (32/36)	95% (36/38)	ns
Spironolactone	93% (69/74)	92% (32/36)	97% (37/38)	ns
Digoxin	39% (29/74)	50% (18/36)	29% (11/38)	0.064
Diuretics	88% (65/74)	86% (31/36)	90% (34-38)	ns
Vasodilators	51% (38/74)	58% (21/36)	45% (17/38)	ns
Patients with target doses of medication according guidelines (%)	73%	77%	63%	<0.001

IQR, interquartile range; Specialized HF care considered (in-hospital or outpatient);HF, heart failure; HF-DMP, disease management program for HF; Centers with at least one multidisciplinary member , centers having at least one of the following professions: cardiology, nursing, physiotherapy and/or physical educator or nutritionist; HF, heart failure; BNP, brain natriuretic peptide; DEIC, Heart Failure Department of the Brazilian Society of Cardiology; ESC, European Society of Cardiology; AHA, American Heart Association; MDP, multidisciplinary program; KCCQ, Kansas City Cardiomyopathy Questionnaire; MLHFQ, Minnesota Living with Heart Failure questionnaire; ACE-I Angiotensin-converting enzyme inhibitors; ARBs, angiotensin receptor blockers; p, public *versus* private.

## APPENDIX Supplementary Table S1 - Questionnaire

I. Site data

1. Institution’s Name: *City UF [Federative Unit] / State:*
2. Type of Service: (Public) / (Private)
  *Note: even if you attend different services, please fulfill this questionnaire according to the type of service you selected (public or private). If you prefer, one can fulfill a second questionnaire with the information of the other service.*

II. Physical and professional structure of the customer service for Heart Failure

1. Approximate number of patients with HF under follow-up: ( )
2. Approximate number of patients with HF attended per week: ( )
3. What is the average monthly number of hospitalizations due to HF in the service?
4. Regarding the outpatient structure, which items are present in your service (select the most applicable item)?
  □General outpatient unit
  □Cardiology outpatient unit
  □HF subspecialized outpatient unit
  □HF subspecialized outpatient and hospitalization units
5. In what way is the outpatient service structured?
  □Hospital outpatient facilities/services
  □Private practice
  □Both (hospital outpatient facility/service and private practice)
6. What are the types of treatment available in your service (check all applicable types):
  □Clinical treatment
  □ICD (implantable cardioverter defibrillator) and Resynchronizer
  □Surgical treatment, except for transplantation
  □Surgical treatment and transplantation
  □Circulatory support
7. Does the service have multiprofessional care? (Yes/No)
8. If it has a multiprofessional service, check which ones and how many professionals in each area.

Cardiology: N=□

Nursing, N=□

Psychology, N=□

Nutrition, N=□

Physiotherapy, N=□

Pharmacist, N=□

Nephrology, N=□

Cardiac Surgeon, N=□

Exercise Physical Educator/Physiotherapist, N=□

Social worker, N=□

Other, N=□

III. Patients and the disease

1. According to your perception, what is the approximate percentage of patients in NYHA functional classes?
  - Functional class I : __%
  - Functional class II : __%
  - Functional class III : __%
  - Functional class IV : __%
2. According to your perception, what is the approximate percentage of the main HF etiologies of patients in your service?
  - Ischemic: __%
  - Valvulopathies __%
  - Hypertensive: __%
  - Chagas Disease: __%
  - Idiopathic: __%
  - Peripartum: __%
  - Cardiotoxicity: __%
3. Other cardiomyopathies: __%
  □Is BNP used in your service? (select all applicable items)
  □No because we do not have access.
  □No because it is not considered essential.
  □Yes, to help in the diagnosis.
  □Yes, to determine the prognosis.
  □Yes, to help define the treatment.
  □Yes, to monitor the patient.
4. In general, who has been initiating HF treatment in your patients? (enter an estimated percentage)
  □Hospital cardiologist (___%)
  □Outpatient facility/practice cardiologist (___%)
  □General practitioner (___%)
  □Intensive care physician (___%)
  □Geriatrician (___%)
  □Other specialties (nephrologist, endocrinologist, emergency physician, other) (___%)
5. What are the most important criteria for assessing disease progression? Number in order of importance (1 being the most important and 6 being the less important; number as “zero” if you do not consider it important).
  □Clinical signs and symptoms
  □Biomarkers (BNP, NT-proBNP)
  □Renal function
  □Oxygen saturation (arterial blood gas)
  □Hemodynamic stability
  □Other

IV. Heart Failure Treatment

1. What is your goal in the treatment of HF patients?
  Number in order of importance (1 being the most important and 5 being the less important).
  □Alleviate HF signs and symptoms (*e.g., edema, dyspnea, etc.)*
  □Prevent hospitalization
  □Improve survival rate
  □Improve quality of life
  □Other
2. What are the greatest medical problems of HF patients despite the currently available treatments?
  Number in order of importance (1 being the most important and 8 being the less important; number as “zero” if you do not consider it important).
  □Drug treatment compliance
  □Non-drug treatment compliance (non-drug actions)
  □Tolerability
  □High mortality rate
  □Low quality of life
  □High rehospitalization rate
  □Non-control of symptoms
  □Loss of renal function
3. What is the most commonly used base treatment for HF patients? (Select all applicable items)
  □ ACE inhibitor □ ARB □ Beta Blocker □ Spironolactone □ Digoxin □ Diuretics □ Vasodilators
4. What is the percentage of HF patients receiving the target dose based on the guideline recommendations?
  (___%) (*numerical field)*
  □ I cannot estimate
5. In which conditions would you consider changing the base conduct of a HF patient?
  Number in order of importance (1 being the most important and 4 being the less important; number as “zero” the situations that you would not change the treatment).
  □On a routine appointment (since it is a progressive disease).
  □When the patient was hospitalized.
  □After hospitalization, the patient is discharged from the hospital and returns to the practice.
  □When the diagnosis indicates worsening disease and/or symptoms.
6. Are there educational programs about the disease, such as classes and lectures, for patients? □ yes □ no
7. Are there educational programs about the disease, such as classes and lectures, for caregivers? □ yes □ no
8. Is instruction given regarding written discharge for patients and/or caregivers? □ yes □ no
9. If yes, who gives this instruction?
  □Nursing
  □Pharmacist
  □Physician responsible for discharge
  □Other
10. During patient instruction, which of the instructions below you consider more difficult to provide (*e.g., due to lack of time or patient acceptance):*
  Select all the applicable items.
  □Instruction about diet
  □Instruction about the need to take the medications regularly
  □Instruction about physical activities
  □Instruction about warning signs and worsening symptoms
  □Instruction about weight increase
  □Instruction about smoking cessation
  □Instruction about future appointments.
11. Regarding patient monitoring programs:
  □Not available
  □An educational program
  □There is a “non-medical” outpatient specialized monitoring program.
  □There is a remote monitoring program via telephone or another appropriate method.
  □There is a home monitoring program.
12. Does the service follow any guidelines? (select only the relevant items that encompass the entire service)
  □The service does not follow a systematic and regular basis.
  □ESC
  □AHA
  □DEIC
  □Assistance intra-institutional protocol (institution’s own protocol)
  □Other
13. Is any questionnaire on the quality of life regularly used?
  □KCCQ
  □Minnesota
  □Other
  □None
14. Is there a cardiac rehabilitation service? □ Yes □ No
15. What are the key performance indicators related to HF in your service? (checking all applicable items)
  □I do not know
  □There are no objective key performance indicators at the moment in my service
  □Hospital mortality rate
  □Mortality rate after six months
  □Rehospitalization rate after 30 days
  □Rehospitalization rate after 90 days
  □Hospital length of stay
  □Other
16. Who determines the conduct of a patient with acute heart failure (AHF) during hospitalization? (*e.g., treatment regimen)*
  Please estimate the percent (*numerical field).*
  □Hospital cardiology team: ___%
  □Cardiologist following the patient in the outpatient facility: ___%
  □Intensive care physician (while the patient is in the ICU): ___%
  (for physicians) Would you like to be mentioned in a potential future publication (as acknowledgements)? If yes, please provide the information below.
  Name: _____ (*free field); Email: _____ (free field).*

**Supplementary Table S2:** Delivered care for heart failure patients in participating centers according to availability of multidisciplinary care.

	Multidisciplinary Care		
	Not Available (n=23)	Available (n=64)	*p*-value
BNP dosage	70% (14/20)	84% (48/57)	ns
Centers with BNP			
Help in the diagnosis	79% (11/14)	69% (33/48)	ns
Determine the prognosis	71% (10/14)	56% (27/48)	ns
Help the treatment	7% (1/14)	42% (20/48)	0.023
Monitoring	57% (8/14)	60% (29/48)	ns
Centers with no BNP			
Lack of access	83% (5/6)	89% (8/9)	ns
Considered not essential	16% (1/6)	22% (2/9)	ns
Education program for patient	6% (1/18)	42% (22/53)	0.004
Education program for caregivers	11% (2/9)	28% (15/53)	ns
Written instructions at hospital discharge	68% (13/19)	89% (48/54)	0.038
By Doctor	62% (10/11)	58% (26/45)	ns
By Nurse	38%	40%	
By Others	0% (0/11)	2% (1/45)	
Patient monitoring programs	6% (1/16)	49% (25/51)	0.001
Using monitoring program			
At ambulatory using non-doctorspecialized monitoring	0% (0/17)	26% (13/51)	0.028
At distance using phone or other method	6% (1/17)	24% (9/51)	ns
Regular use of HF guidelines	83% (15/18)	92% (48/52)	ns
DEIC	61% (11/18)	38% (20/52)	ns
ESC	5% (1/18)	19% (10/52)	
AHA	17% (3/18)	21% (11/52)	
Institution's own protocol	0% (0/18)	12% (6/52)	
Other guidelines	0% (0/18)	2% (1/52)	
Not following guidelines	17% (3/18)	8% (4/52)	
Using quality of life questionnaire	35% (6/17)	24% (12/50)	ns
KCCQ	3% (1/17)	3% (3/50)	ns
MLHFQ	29% (5/17) (5 out of 17)	18% (9/50) (9 out of 50)	ns
Other	0% (0/17)	4% (2/50)	ns
Cardiac rehabilitation	17% (3/18)	69% (33/50)	<0.001
Key performance indicators	40% (6/15)	55% (26/47)	ns
Hospital mortality	27% (4/15)	32% (15/47)	ns
6-month mortality	13% (2/15)	21% (10/47)	ns
30-day hospitalization after discharge	13% (2/15)	38% (18/47)	ns
90-day hospitalization after discharge	7% (1/15)	17% (8/47)	ns
Hospitalization duration	7% (1/15)	30% (14/47)	0.09
Other	0% (0/17)	4% (2/47)	ns
Medical decision in hospitalized patient in decompensated HF			
By hospital cardiology team	76%	44%	<0.001
By cardiologist who cares for the patient in the outpatient clinic	18%	43%	<0.001
By intensive care unit doctors if admitted in intensive care unit	6%	13%	0.023
Beginning of HF treatment			
Cardiologist in hospital	40%	38%	ns
Cardiologist in ambulatory	43%	42%	ns
General practitioner	5%	11%	0.078
Intensive care doctor	6%	4%	ns
Geriatrics	2%	2%	ns
Others	4%	3%	ns
Prescribed HF treatment			
ACE-I	100% (18/18)	94% (51/54)	ns
ARBs	78% (14/18)	87% (47/64)	ns
Β-blocker	89% (16/18)	93% (50/54)	ns
Spironolactone	100% (15/18)	93% (50/54)	ns
Digoxin	33% (6/18)	43% (23/54)	ns
Diuretics	78% (14/18)	91% (49/54)	ns
Vasodilators	55% (10/18)	52% (28/54)	ns
Patients with target doses of medication according guidelines (%)	45%	61%	<0.001

IQR, interquartile range; Specialized HF care considered (in-hospital or outpatient); HF, heart failure; HF-DMP, Disease Management Program for HF; Centers with at least one multidisciplinary member, centers having at least one of the following professions: cardiology, nursing, physiotherapy and/or physical educator or nutritionist; HF, heart failure; BNP, brain natriuretic peptide; DEIC, Heart Failure Department of the Brazilian Society of Cardiology; ESC, European Society of Cardiology; AHA, American Heart Association; MDP, multidisciplinary program; KCCQ, Kansas City Cardiomyopathy Questionnaire; MLHFQ, Minnesota Living with Heart Failure questionnaire; ACE-I Angiotensin-converting enzyme inhibitors; ARBs, angiotensin receptor blockers.

**Supplementary Table S3:** Delivered care for heart failure patients in participating centers according to heart failure subspecialized care.

	HF Subspecialized Care		
	Not Available (n=48)	Available (n=42)	*p*-value
BNP dosage	65% (31/48)	58% (33/57)	ns
Centers with BNP			
Help in the diagnosis	71% (22/31)	73% (24/33)	ns
Determine the prognosis	65% (20/31)	58% (19/33)	ns
Help the treatment	42% (13/31)	24% (8/33)	ns
Monitoring	62% (19/31)	55% (18/33)	ns
Centers with no BNP			
Lack of access	100% (7/7)	75% (6/8)	ns
Considered not essential	0% (0/6)	38% (3/8)	ns
Education program for patient	53% (18/34)	41% (16/39)	ns
Education program for caregivers	17% (6/35)	31% (12/39)	ns
Written instructions at hospital discharge	77% (27/35)	90% (36/40)	ns
By Doctor	79% (19/24)	56% (19/34)	ns
By Nurse	21% (5/24)	41% (14/34)	
By Others	0% (0/24)	2.9% (1/34)	
Patient monitoring programs	23% (7/31)	51% (20/39)	0.014
Using monitoring program			
At ambulatory using non-doctorspecialized monitoring	3.2% (1/31)	33% (13/39)	0.002
At distance using phone or other methods	10% (3/31)	18% (7/39)	ns
Regular use of HF guidelines	84% (27/32)	95% (38/40)	ns
DEIC	52% (14/27)	45% (17/38)	ns
ESC	26% (7/27)	21% (8/38)	
AHA	19% (5/27)	18% (7/38)	
Institution's own protocol	3.7% (1/27)	13% (5/38)	
Other guidelines	0% (0/27) out of 27)	2.6% (1/38)	
Not following guidelines	19% (5/27)	5.3% (2/38)	
Using quality of life questionnaire	23% (7/31)	29% (11/38)	ns
KCCQ	6.5% (2/31)	5.3% (2/38)	ns
MLHFQ	16% (5/31)	24% (9/38)	ns
Other	3.2% (1/31)	2.6% (1/38)	ns
Cardiac rehabilitation	27% (8/30)	76% (29/38)	<0.001
Key performance indicators	50% (14/28)	56% (20/36)	ns
Hospital mortality	25% (7/28)	36% (13/36)	ns
6-month mortality	18% (5/28)	22% (8/36)	ns
30-day hospitalization after discharge	25% (7/28)	39% (14/36)	ns
90-day hospitalization after discharge	4% (1/28)	22% (8/36)	0.066
Hospitalization duration	18% (5/28)	30% (11/36) (11 out of 36)	ns
Other	0% (0/28)	6% (2/36)	ns
Medical decision in hospitalized patient in decompensated HF			
By hospital cardiology team	41%	45%	<0.001
By cardiologist who cares for the patient in the outpatient clinic	45%	42%	<0.001
By intensive care unit doctors if admitted in intensive care unit	14%	12%	0.067
Beginning of HF treatment			
Cardiologist in hospital	41%	45%	<0.001
Cardiologist in ambulatory	41%	31%	<0.001
General practitioner	6%	14%	<0.001
Intensive care doctor	9%	3%	<0.001
Geriatrics	1%	2%	ns
Others	2%	4%	ns
Prescribed HF treatment			
ACE-I	97% (33/34)	95% (38/40)	ns
ARBs	79% (27/34)	88% (35/40)	ns
Β-blocker	97% (33/34)	88% (35/40)	ns
Spironolactone	91% (31/34)	95% (38/40)	ns
Digoxin	24% (8/34)	53% (21/40)	0.017
Diuretics	82% (28/34)	93% (37/40)	ns
Vasodilators	34% (13/34)	63% (25/40)	0.037
Patients with target doses of medication according guidelines (%)	46%	68%	<0.001

IQR, interquartile range; Specialized HF care considered (in-hospital or outpatient); HF, heart failure; HF-DMP, Disease Management Program for HF; Centers with at least one multidisciplinary member, centers having at least one of the following professions: cardiology, nursing, physiotherapy and/or physical educator or nutritionist; HF, heart failure; BNP, brain natriuretic peptide; DEIC, Heart Failure Department of the Brazilian Society of Cardiology; ESC, European Society of Cardiology; AHA, American Heart Association; MDP, multidisciplinary program; KCCQ, Kansas City Cardiomyopathy Questionnaire; MLHFQ, Minnesota Living with Heart Failure questionnaire; ACE-I Angiotensin-converting enzyme inhibitors; ARBs, angiotensin receptor blockers.

**Supplementary Table S4:** Delivered care for heart failure patients in participating centers comparing centers according to higher specialized multidisciplinary care.

	HF higher specialized multidisciplinary care		
	Not Available (n=23)	Available (n =42 )	*p*-value
BNP dosage	70% (14/20)	90% (37/41)	0.066
Centers with BNP			
Help in the diagnosis	79% (11/14)	70% (26/37)	ns
Determine the prognosis	71% (10/14)	60% (22/37)	ns
Help the treatment	7% (1/14)	38% (14/37)	0.041
Monitoring	57% (8/14)	60% (22/37)	ns
Centers with no BNP			
Lack of access	100% (7/7)	75% (6/8)	ns
Considered not essential	0% (0/6)	38% (3/8)	ns
Education program for patient	53% (18/34)	41% (16/39)	ns
Education program for caregivers	17% (6/35)	31% (12/39)	ns
Written instructions at hospital discharge	77% (27/35)	90% (36/40)	ns
By Doctor	79% (19/24)	56% (19/34)	Ns
By Nurse	21% (5/24)	41% (14/34)	
By Others	0% (0/24)	3% (1/34)	
Patient monitoring programs	23% (7/31)	51% (20/39)	0.014
Using monitoring program			
At ambulatory using non-doctorspecialized monitoring	3% (3/11)(1 out of 31)	33% (13/39)(13 out of 39)	0.002
At distance using phone or other method	10% (3/31)	18% (7/39)	ns
Regular use of HF guidelines	84% (27/32)	95% (38/40)	ns
DEIC	52% (14/27)	45% (17/38)	ns
ESC	26% (7/27)	21% (8/38)	
AHA	19% (5/27)	18% (7/38)	
Institution's own protocol	3% (1/27)	13% (5/38)	
Other guidelines	0% (0/27)	2.6%(1/38)	
Not following guidelines	19% (5/27)	5% (2/38)	
Using quality of life questionnaire	23% (7/31)	29% (11/38)	ns
KCCQ	7% (2/31)	5% (2/38)	ns
MLHFQ	16% (5/31)	24% (9/38)	ns
Other	3% (1/31)	3% (1/38)	ns
Cardiac rehabilitation	27% (8/30)	76% (29/38)	<0.001
Key performance indicators	50% (14/28)	56% (20/36)	ns
Hospital mortality	25% (7/28)	36% (13/36)	ns
6-month mortality	18% (5/28)	22% (8/36)	ns
30-day hospitalization after discharge	25% (7/28)	39% (14/36)	ns
90-day hospitalization after discharge	4% (1/28)	22% (8/36)	ns
Hospitalization duration	18% (5/28)	31% (11/36)	ns
Other	0% (0/28)	6% (2/36)	ns
Medical decision in hospitalized patient in decompensated HF			
By hospital cardiology team	42%	33%	<0.001
By cardiologist who cares for the patient in the outpatient clinic	45%	57%	<0.001
By intensive care unit doctors if admitted in intensive care unit	13%	10%	0.002
Beginning of HF treatment			
Cardiologist in hospital	40%	45%	0.009
Cardiologist in ambulatory	43%	27%	<0.001
General practitioner	5%	16%	0.006
Intensive care doctor	6%	5%	ns
Geriatrics	2%	2%	ns
Others	4%	5%	ns
Prescribed HF treatment			
ACE-I	97% (33/34)	95% (38/40)	ns
ARBs	79% (27/34)	88% (35/40)	ns
Β-blocker	97% (33/34)	88% (35/40)	ns
Spironolactone	91% (31/34)	95% (38/40)	ns
Digoxin	24% (8/34)	53% (21/40)	0.017
Diuretics	82% (28/34)	93% (37/40)	ns
Vasodilators	38% (13/34)	63% (25/40)	0.037
Patients with target doses of medication according guidelines (%)	45%	65%	<0.001

IQR, interquartile range; Specialized HF care considered (in-hospital or outpatient); HF, heart failure; HF-DMP, Disease Management Program for HF; Centers with at least one multidisciplinary member, centers having at least one of the following professions: cardiology, nursing, physiotherapy and/or physical educator or nutritionist; HF, heart failure; BNP, brain natriuretic peptide; DEIC, Heart Failure Department of the Brazilian Society of Cardiology; ESC, European Society of Cardiology; AHA, American Heart Association; MDP, multidisciplinary program; KCCQ, Kansas City Cardiomyopathy Questionnaire; MLHFQ, Minnesota Living with Heart Failure questionnaire; ACE-I Angiotensin-converting enzyme inhibitors; ARBs, angiotensin receptor blockers.
